# Effectiveness of different types of skin grafting for treating venous leg ulcers: A protocol for systematic review and network meta-analysis

**DOI:** 10.1097/MD.0000000000025597

**Published:** 2021-04-16

**Authors:** Junqing Pan, Xiangjun Hu, Hongwei Yin, Congzhong Zhang, Zhangren Yan

**Affiliations:** a Jiangxi University of Traditional Chinese Medicine; b Affiliated Hospital of Jiangxi University of Traditional Chinese Medicine, Nanchang, Jiangxi Province, China.

**Keywords:** network meta-analysis, protocol, skin grafting, venous leg ulcers

## Abstract

**Background:**

Venous leg ulcers (VLUs) are the most common ulcer on the lower extremity, with 4% of patients over the age of 65 suffering from VLUs worldwide. As a recurrent, chronic, disabling disease, VLUs are associated with prolonged disability, substantial socioeconomic impact, and significant psychosocial morbidity. At present, Skin grating is one of the most effective treatments for non-healing ulcers. However, there are still no new studies based on the latest research and new research methods to evaluate and compare the effect of different types of skin grafts for treating venous leg ulcers. Therefore, a Bayesian network meta-analysis (NMA) will be conducted to systematically assess skin grafting efficacy for VLUs.

**Methods:**

We will include randomized controlled trials (RCTs) involving patients with VLUs treated by skin grafts. Electronic databases and clinical trials registries will be searched from their inception until June 2021, without language or publication restrictions on status. The search strategy mainly includes Medical Subject Headings (MeSH) and free-text terms. Two review authors will independently perform data extraction and assessment of study quality. And We will use Bayesian NMA to evaluate all available evidence in STATA 14.0 and WinBUGS software.

**Results:**

This protocol will use Bayesian NMA to assess the effectiveness of different types of skin grafting for treating venous leg ulcers.

**Conclusion:**

This study aims to synthesize the available evidence from RCTs in a network meta-analysis to summarize the best research available and provide consistency among treatment protocols given to patients, resulting in improved efficacy and the quality of care and reduced cost.

## 1 Introduction

Venous leg ulcer(VLUs), open skin lesion of the leg or foot, occur in an area affected by venous hypertension.^[1]^ VLUs are the most prevalent ulcer on the lower extremity, accounting for 70% of all leg ulcers, affecting many individuals worldwide.^[2]^ Annual prevalence rates of VLUs per 1000 population ranged from 4.5 in India, 1.7 in China, 1.5 in Brazil, and 1.2 in Australia.^[3–6]^ And VLUs have a higher incidence with age, the prevalence of VLUs is up to 2% of the population, increases to 5% of patients over 65 years of age.^[7,8]^ With the rapid increase in the aging population, VLUs will become an increasing burden for health care expenditures. What's more, as a recurrent, chronic, disabling disease, VLUs are associated with protracted disability, huge medical costs, and significant psychosocial morbidity.^[9]^ The annual cost per VLUs patient in the UK was estimated at £10,000–30,000 per year.^[10]^ Hence, for VLUs, it is necessary to explore a clinically effective treatment method that can improve wound-healing rates and the quality of life, reduce the recurrence rates and medical costs.

As a time-tested approach, skin grafting dates back to Hindu surgeons circa 800 BCE who used to repair the nasal mutilations of individuals punished, and the basic principles of grafting have carried into the modern era.^[11]^ Today, skin grafting is one of the most common and effective treatments for non-healing ulcers. One high-quality Cochrane review included 17 trials (1034 participants) and a systematic review in evidence-based medicine concluded that significantly more ulcers healed when treated with bilayer artificial skin.^[12,13]^ And Clinical practice guidelines of the Society for Vascular Surgery and the American Venous Forum recommend that skin grafting and cell-based therapies represent a second-line strategy when a minimum of 4 to 6 weeks of standard wound therapy fails.^[9]^ Nonetheless, insufficient evidence to determine whether others skin grafting types increased the healing of venous ulcers.^[12]^ Nowadays, with the development of skin grafting technology and cell regeneration medicine, various skin grafting techniques have been applied in clinical practice, especially in wound healing. There are still no new studies based on the latest trials and new research methods to evaluate and compare different skin grafting effects for VLUs. Further studies are required to assess whether other forms of skin grafting increase ulcer healing.

To address these problems, we designed a new protocol that would update new evidence and redesigned the search strategy. Furthermore, an advanced meta-analysis technique, network meta-analysis, be adopted. This approach can simultaneously compare multiple competing interventions in a single statistical model while maintaining randomization as with standard meta-analysis.^[14,15]^ This study aims to synthesis the available evidence from RCTs in a network meta-analysis to summarize the best research available, assess the effect of different type of skin grafting in the treatment of VLUs, provides consistency among treatment protocols given to patients, resulting in improved efficacy and the quality of care and reduced cost.

## 2 Protocol registration

This protocol will be conducted and reported by the guideline of the Preferred Reporting Items for Systematic Reviews and Meta-Analysis Protocols (PRISMA-P) and the PRISMA Extension Statement.^[16,17]^ This study protocol has been registered on International Platform of Registered Systematic Review and Meta-analysis Protocols website (INPLASY202130093).

## 3 Methods

### 3.1 Eligibility criteria

#### 3.1.1 Study type

We will include published and unpublished Randomized controlled trials (RCTs). No date or language restrictions will be applied.

#### 3.1.2 Participants

We will include RCTs involving participants in any care setting with VLUs. No further restrictions will be made on participants’ age, gender, ethnicity, and nationality. Methods to diagnose VLUs wounds may vary and this review will accept any as described by the included studies. Some trials would be included that included participants with arterial, mixed, neuropathic, and diabetic ulcers only if the outcomes for those with VLUs were reported separately.

#### 3.1.3 Interventions

The primary intervention was skin grafts or skin replacements applied to treat VLUs. We included studies which compared the following types of grafts with any other intervention:

(1) pinch grafts (autografts),
(2) split-thickness grafts (autografts),
(3) full-thickness grafts (autografts and xenografts),
(4) cultured keratinocytes/epidermal grafts (allografts and xenografts),
(5) artificial skin, bioengineered skin equivalents (allografts and xenografts),

#### 3.1.4 Outcome indicators

##### 3.1.4.1 Primary outcomes

The primary outcome for this review is complete wound healing. A trial had to report at least one of the following as providing the most relevant measures of outcome for the analyses:

(1) objective measures of healing (Change or rate of change in wound size, with adjustment),
(2) time to complete healing,
(3) proportion of ulcers healed within the trial period, as defined by the trial authors,
(4) recurrence of VLUs (as reported in the trial).

##### 3.1.4.2 Secondary outcomes

Secondary outcomes included: Health-related quality of life; pain; adverse events; costs withdrawals and acceptability of treatment.

### 3.2 Database and search strategy

Studies search will conduct on the following electronic databases: Cumulative Index to Nursing and Allied Health Literature (EBSCO CINAHL Plus), the Cochrane Library, Web of Science, PubMed, Ovid MEDLINE; Ovid EMBASE, China BioMedical Literature (CBM), China National Knowledge Infrchrome1astructure (CNKI), and Wanfang database. In addition, we will search clinical trials registries: the Cochrane Central Register of Controlled Trialsx, the Cochrane Wounds Specialised Register, US National Institutes of HealthOngoing Trials Register Clinical Trials, World Health Organization (WHO) International Clinical Trials Registry Platform (ICTRP), EU Clinical Trials Register. All the databases will be searched from their inception until June 2021, without restrictions for language, or publication on status. The search strategy mainly includes Medical Subject Headings (MeSH) and free-text terms. The detailed search strategy of PubMed is shown in Table 1.

**Table 1:** Detailed search strategy for PubMed.

Number	Search item
1#	“Leg Ulcer+”[MesHTerms]
2#	“varicose ulcer^∗^”[Title/Abstract] OR “venous ulcer^∗^”[Title/Abstract] OR “leg ulcer^∗^ “[Title/Abstract] OR “foot ulcer^∗^”[Title/Abstract] OR “feet N1 ulcer^∗^ “[Title/Abstract] OR”stasis ulcer^∗^”[Title/Abstract] OR “crural ulcer^∗^”[Title/Abstract] OR” lower extremity N3 ulcer^∗^”[Title/Abstract]
3#	1# 0R 2#
4#	“Skin Transplantation”[MeSH Terms]
5#	“skin graft^∗^”[Title/Abstract] OR “pinch graft^∗^”[Title/Abstract] OR “split thickness “[Title/Abstract] OR “full thickness”[Title/Abstract] OR “allograft^∗^^∗^ “[Title/Abstract] OR”dermagraft^∗^”[Title/Abstract] OR “apligraf^∗^”[Title/Abstract]
6#	“tissue N2 engineer^∗^”[Title/Abstract] OR “cultured N2 keratinocyte^∗^”[Title/Abstract] OR “human-tissue graft”[Title/Abstract] OR “bioengineered skin”[Title/Abstract] OR “human-skin device “[Title/Abstract] OR “living-skine quivalent”[Title/Abstract] OR “artificial skin “[Title/Abstract] OR “ skin substitutes”[Title/Abstract] OR
7#	4# OR 5# OR 6#
8#	“randomized controlled trial”[Title/Abstract] OR “controlled clinical trial” [Title/ Abstract] OR “Randomized”[Title/Abstract] OR “random allocation”[Title/Abstract] OR “Randomly”[Title/Abstract]
9#	3# AND 7# AND 8#

### 3.3 Study selection and data extraction

Two review authors will independently assess the titles and abstracts of the search strategy's results in terms of their relevance and design and then inspect the full text of all potentially eligible studies according to the eligibility criteria. The Cochrane review will be checked again for relevance.

We will establish the document information extraction table in pre-designed Excel. Two review authors will independently extract the following information from each included study: article title, author, publication time, demographic characteristics of the subjects, sample size, allocation method, allocated to intervention and control groups, course of treatment, the severity of disease, adverse events, data analysis strategy, and outcome indicators. We will record the reasons for the excluded studies. The results extracted by two review authors will be cross-checked. Disagreements will be resolved by discussion and, where required, the input of a third review author.

### 3.4 Risk assessment of bias and quality assessment

Two review authors will independently use Cochrane's Risk of Bias tool to appraise the risk of bias of each included study.^[18]^ Any discrepancy between two reviewers will be resolved by discussion and a third reviewer where necessary. Meanwhile, we followed the Grading of Recommendations Assessment Development and Evaluation (GRADE) approach proposed by Salanti and colleagues to assess the certainty of evidence from the network meta-analysis for each network contrast and the ranking of intervention groups: the overall certainty could be rated from high, moderate, low to very low.^[19]^

### 3.5 Data analysis

Firstly, data will be synthesized with a pairwise meta-analysis in a frequentist framework by RevMan (version 5.3, Cochrane). Risk ratio and 95% confidence interval (CI) will be used for dichotomous outcomes, mean differences or standardized mean differences with 95% CI will be used for continuous outcomes. Cochrane *Q* test and *I*^*2*^ test will be used for heterogeneity assessment.^[20]^ If no significant statistical heterogeneity exists or heterogeneity is small (*P* ≥ .10 and *I*^*2*^ ≤ 50%), the Mantel-–Haenszel fixed effect model will be employed, otherwise using a random effects model (*P* < .10 and *I*^*2*^ > 50%).

Then, we will conduct Bayesian NMAs to compare the efficacy of different types of skin grafting in the treatment of VLUs. Markov Chains Monte Carlo method will be conducted in the WinBUGS software (Version 1.43, Medical Research Council Biostatistics Unit, Cambridge, UK). Four chains are used for simulation. We will set the number of iterations to 50,000, use the first 20,000 annealing times to eliminate the influence of the initial value, and set the step length to10.^[21]^ Meanwhile, the potential scale reduction parameter (potential scale reduced factor, PSRF) is used to evaluate the convergence of the results. When the PSRF is close to 1, it indicates that the results have good convergence and the obtained results are highly reliable.^[22]^ We also calculate the relative ranking of the various skin grafting based on the surface under the cumulative ranking curves (SUCRA) percentages range from 0 to 1, with 1 indicating that treatment is sure to be the best and 0 that treatment is certain to be the worst. The area under the curve increases as the SUCRA value increases, indicating that the intervention is more effective.^[19]^

### 3.6 Assessment of heterogeneity, sensitivity analysis, subgroup analysis

For the test results with obvious heterogeneity, we will further conduct sensitivity analysis to explore the source of heterogeneity, and carry out subgroup analysis according to the different heterogeneity sources, such as treatment time, course of disease, basic disease, gender, age, and so on. If there is no clear source of heterogeneity, only descriptive analysis can be carried out.

### 3.7 Assessment of inconsistency

Skin grafting can be classified into multiple types, and there are various of intervention measures for VLUs in studies. In the direct evidence and indirect evidence network of each outcome indicator, the node-splitting model will be used to test inconsistency in every closed loop of evidence through Stata software.^[23]^ If *P* > .05, the consistency model is adopted; otherwise, the consistency model is adopted. For the consistency model analysis results, the inconsistent model's stability can be tested by the inconsistent model when the inconsistent model factors include 0 and the standard inconsistency deviation. When the consistent effect model's random standard deviation is approximately equal to the standard deviation of the inconsistent model, the consistency model results are more stable and reliable.

### 3.8 Assessment of publication bias

We will assess the publication bias plotting a comparison-adjusted funnel plot for the network,^[24,25]^ and statistically using two formal tests, Begg rank correlation test^[26]^ and Egger regression asymmetry test, which detect asymmetry of funnel plots.^[27]^ Comparison-specific risk of bias will be appraised for each valid direct comparison of wound healing rates.^[25]^ Two-sided *P* < .05 will be considered statistically significant. If the funnel plot shows asymmetry or distribution difference, it indicates publication bias or a small sample effect.

### 3.9 Ethics and dissemination

All data in the conception and design of this protocol are from published studies and do not involve patients and the public, so ethical approval is not required. The results of the study will be submitted to a peer-reviewed journal for publication.

## 4 Discussion

As a standard method of clinical wound repair, skin grafting is developing continuously. The emergence and clinical application of stem cells, bioengineered skin equivalents, Cell-based skin therapies, and genetically modified tissue transplantation artificial skin have provided new options for skin transplantation and made the clinical decision difficult. Previous studies have evaluated the efficacy of different types of skin grafts in the repair of venous ulcers. However, with the application of new skin grafts and the production of new research results, we need to redesign the program to evaluate the efficacy of skin grafts in the repair of venous ulcers. Therefore, we will use network meta-analysis to compare several different skin grafting types and evaluate the effectiveness, to provide evidence-based medicine for clinical decision-makers.

## Author contributions

**Conceptualization:** Junqing Pan, Zhangren Yan.

**Methodology:** Junqing Pan, Xiangjun Hu, Hongwei Yin.

**Project administration:** Zhangren Yan.

**Software:** Congzhong Zhang, Xiangjun Hu.

**Supervision:** Zhangren Yan.

**Writing – original draft:** Junqing Pan.

**Writing – review & editing:** Junqing Pan, Zhangren Yan.
